# Phytochemical Characterization, In Vitro Anti-Inflammatory, Anti-Diabetic, and Cytotoxic Activities of the Edible Aromatic Plant; *Pulicaria jaubertii*

**DOI:** 10.3390/molecules26010203

**Published:** 2021-01-03

**Authors:** Hamdoon A. Mohammed, Mohammed F. Abdelwahab, El-Sayed M. El-Ghaly, Ehab A. Ragab

**Affiliations:** 1 Department of Medicinal Chemistry and Pharmacognosy, College of Pharmacy, Qassim University, Buraydah 51452, Saudi Arabia; 2 Department of Pharmacognosy, Faculty of Pharmacy, Al-Azhar University, Cairo 11371, Egypt; mohabdelwahab@yahoo.com (M.F.A.); sayedghaly86@yahoo.com (E.-S.M.E.-G.); ar_ehab@yahoo.com (E.A.R.)

**Keywords:** *Pulicaria jaubertii*, triterpenes, anti-inflammatory, anti-diabetic, cytotoxicity

## Abstract

*Pulicaria jaubertii* is a medicinal herb that alleviates inflammations and fever. Chromatographic separation, phytochemical characterization, and in vitro biological activities of the plant *n*-hexane extract were conducted for the first time in this study. Six compounds were isolated for the first time from the *n*-hexane fraction of *Pulicaria jaubertii* aerial parts and were identified on the bases of NMR and MS analyses as pseudo-taraxaterol (**1**), pseudo-taraxasterol acetate (**2**), 3β-acetoxytaraxaster-20-en-30-aldehyde (**3**), calenduladiol-3-*O*-palmitate (**4**), stigmasterol (**5**), and α-tocospiro B (**6**). Compound (**6**) was a rare tocopherol-related compound and was isolated for the first time from family Asteraceae, while compound (**3**) was isolated for the first time from genus *Pulicaria*. The total alcoholic extract and *n*-hexane fraction were tested for their anti-inflammatory, antidiabetic, and cytotoxic activities. The *n*-hexane fraction has dose dependent red blood cells (RBCs) membrane stabilization and inhibition of histamine release activities with IC_50_: 60.8 and 72.9 µg/mL, respectively. As antidiabetic activity, the alcoholic extract exerted the most inhibition on the activity of yeast α-glucosidase, with an IC_50_: 76.8 µg/mL. The *n*-hexane fraction showed cytotoxic activity against hepatocarcinoma (HepG-2), breast carcinoma (MCF-7), and prostate carcinoma (PC-3) cell lines with IC_50_: 51.8, 90.8 and 62.2 µg/mL, respectively. In conclusion, the anti-inflammatory effect of *Pulicaria jaubertii* might be attributed to the triterpenoid constituents of the *n*-hexane extract of the plant.

## 1. Introduction

Medicinal plants and their products have been used in human life quality improvement [1]. They have been used in disease remediation processes around the world and their natural derived product is still the focus of researchers in the drug discovery and in the areas of biology [1,2,3]. Genus *Pulicaria*, belonging to the Asteraceae family, tribe Inuleae, consists of 100 species distributed in Europe, North Africa, and Asia, especially in Yemen and Saudi Arabia and is known for its medicinal properties [4]. In Yemen and Saudi Arabia, plants of genus *Pulicaria* are known for their common uses as herbal teas, insect repellant, and alleviate inflammations [4]. In addition, antiproliferative and anti-inflammatory activities were also reported for these plants [5]. Earlier phytochemical investigations on different *Pulicaria* species afforded several sesquiterpenoids [6,7], diterpenoids [8], triterpenes [9], flavonoids [10], and phenolics [11,12]. *Pulicaria jaubertii* [syn. *Pulicaria orientalis* Jaub. and Spach] is one of the *Pulicaria* species indigenous in Yemen. It is a perennial aromatic plant used by local people as a flavoring agent for food preparation [5]. The plant is used also in the preparation of soaps and as a spicing agent due to their essential oil constituents [5,6]. In addition, *Pulicaria jaubertii* has an important application in the folk medicine, as it is used for treatment of inflammation, urogenital pyritic conditions, and for relieving fever [5]. In addition, it has been used as a diuretic and insect repellent [5,7,8]. The volatile oil composition of *Pulicaria jaubertii* species originated from different sources was reported. In addition, the antimicrobial, antifungal, antimalarial, and insecticidal activities of the plant were previously investigated [5,7,8]. Flavonols, dihydroflavonols, monoterpene glucosides, stigmasterol glucoside, and hydroquinones were isolated from the ethyl acetate fraction and CH_2_Cl_2_-MeOH extract of *Pulicaria jaubertii* [9,10,11]. Despite the large numbers of the articles dealing with the chemical investigation and constituents’ identification of the *Pulicaria jaubertii*, the nonpolar *n*-hexane fraction has not been chemically inspected. Phytochemical investigation of the plant *n*-hexane extract was conducted in the current study as a part of *Pulicaria jaubertii* phytochemical characterization. Biological investigations in this study were conducted for the total alcoholic and *n*-hexane extracts of the plant and were rationally selected according to the reported traditional use of the plant.

## 2. Results and Discussion

### 2.1. Isolation and Structural Identification of n-hexane Constituents

**Figure 1:**
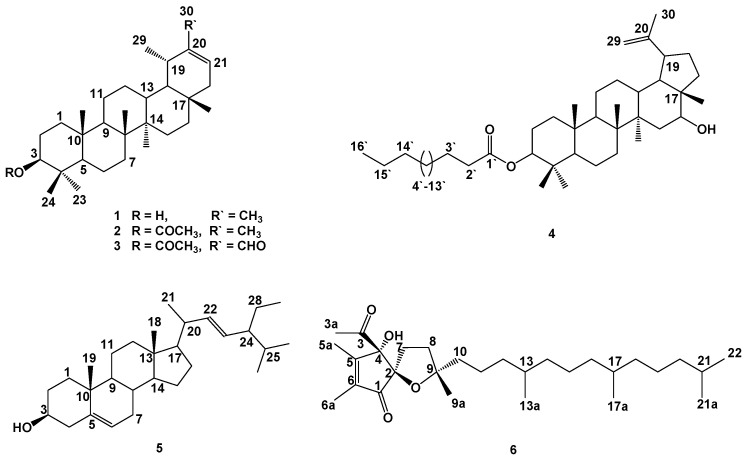
Chemical structures of the isolated compounds (**1**-**6**).

The present work describes the identification of four triterpenes (**1**–**4**), one sterol (**5**) and one tocopherol-related compound (**6**) from the *n*-hexane extract of *Pulicaria jaubertii* aerial parts. The total alcoholic extract of *Pulicaria jaubertii* was fractionated between *n*-hexane, ethyl acetate, and *n*-butanol in sequence. Successive chromatographic work-up on the *n*-hexane soluble part using different techniques, i.e., VLC, solid-phase extraction (RP-C_18_) and open column chromatography gives six compounds (**1**–**6**) (Figure 1). These compounds were identified by analysis of their ^1^H and ^13^C-NMR (Supplementary Materials) and MS spectroscopic data and comparison with those in the literature as pseudo-taraxaterol (**1**) [12,13], pseudo-taraxasterol acetate (**2**) [14], β-acetoxytaraxaster-20-en-30-aldehyde (**3**) [15], calenduladiol-3-*O*- palmitate (**4**) [16,17], stigmasterol (**5**) [18,19], and α-tocospiro B (**6**) [20].

Compound (**1**) was isolated as a white crystalline solid; mp 214–216 °C. The ESI-MS showed a pseudo-molecular ion peak at *m*/*z* 445 [M + H_2_O + H]^+^. The IR spectrum exhibited absorption bands corresponding to a hydroxyl group (3400 cm^−1^) and a trisubstituted double bond (1650 cm^−1^). The ^1^H-NMR spectrum of compound **1** exhibited the signals of seven singlet tertiary methyls at δ 0.73 (Me-28), 0.76 (Me-24), 0.85 (Me-25), 0.95 (Me-27), 0.97 (Me-23), 1.04 (Me-26), and 1.63 (Me-30), one doublet secondary methyl at δ 0.99 (*J.*= 5.9 Hz, Me-29), an oxymethine at δ 3.20 (dd, *J.*= 11.7, 4.5 Hz, H-3α), and an olefinic proton at δ 5.26 (d, *J.*= 6.8 Hz, H-21). The ^13^C-NMR spectrum of **1** displayed 30 carbon resonances, including two olefinic carbons at δ 139.86 (C-20) and 118.87 (C-21) and one oxygen bearing carbon at δ 79.03 (C-3), indicating a pentacyclic triterpene alcohol with one trisubstituted double bond. According to these data and through comparison with the literature [12,13], compound **1** was concluded to be pseudo-taraxasterol.

Compound (**2**) was isolated as colorless needles; mp 239–241 °C. The molecular formula was deduced to be C_32_H_48_O_2_ from EIMS (*m/z* 468) suggesting the presence of an additional acetoxy group in comparison to **1**, which was established by the IR absorption band at 1735 cm^−1^ for an ester group. The ^1^H and ^13^C-NMR spectral data of **2** were almost identical to that of **1** except for ring A. The major differences were the presence of signals corresponding to an acetyl group (δ_H_ 2.04, *s*; δ_C_ 171.06 and 21.35) attached to C-3 position in **2** instead of a hydroxyl group in **1**. The downfield shift of H-3 proton, which appeared at δ 4.48 (dd, *J.*= 11.0, 5.1 Hz, H-3) in **2** compared with the corresponding proton in **1** (δ 3.20), confirmed the presence of the acetyl moiety. The large vicinal coupling constant (11.0 Hz) is consistent with an axial position for H-3 and an equatorial orientation for the acetate. From these data it was evident that compound **2** was the acetylated derivative of pseudo-taraxasterol **1**. On the basis of the abovementioned data and through comparison with the literature [14], compound **2** was identified as pseudo-taraxaterol acetate.

Compound (**3**) was isolated as colorless needles; mp 231–233 °C. The molecular formula was deduced to be C_32_H_46_O_3_ from the observed EIMS molecular ion peak at *m/z* 482 [M]^+^. An ester carbonyl (1733 cm^−1^) and an α,β-unsaturated carbonyl (1692 and 1645 cm^−1^) absorptions were observed in its IR spectrum. It showed an absorption band at 235 nm in the UV spectrum indicated the presence of conjugation in the molecule. The ^1^H and ^13^C-NMR spectral data of **3** were almost identical to that of **2**, suggested the presence of a monounsaturated pentacyclic 3β-acetoxytriterpene of the pseudotaraxasterane skeleton with a methyl group oxidized to aldehyde in **3** versus **2**. The presence of an aldehyde group in the molecule was established from the observed downfield proton signal at δ 9.36 (s, H-30) in the ^1^H-NMR spectrum and the downfield carbon signal at δ 194.11 (C-30) in the ^13^C-NMR spectrum of **3**. Additionally, the downfield shifted proton at δ 6.71 (dd, *J.*= 6.8, 2.5 Hz, H-21) and carbon at δ 149.28 (C-21) compared to those of **1** and **2** confirmed the presence of the aldehyde group at C-20 position. From the above findings, the structure of **3** was assigned to be 3β-acetoxytaraxaster-20-en-30-aldehyde [15].

Compound (**4**) was obtained as white amorphous powder and its molecular formula was determined to be C_46_H_80_O_3_ as indicated from the molecular ion peak at *m/z* 680 in the EIMS spectrum and from ^13^C-NMR spectrum. The IR spectrum exhibited absorption bands at 1730 cm^−1^ for ester carbonyl, 1640 and 883 cm^−1^ for exocyclic di-substituted double bond and 725 cm^−1^ for long aliphatic chain [21,22]. The ^1^H-NMR spectrum of **4** showed the characteristic signals for seven tertiary methyl groups, which are reminiscent of a lupeol-type triterpene at δ 0.79 (s, Me-28), 0.83 (s, Me-23), 0.84 (s, Me-24), 0.85 (s, Me-25), 0.98 (s, Me-27), 1.03 (s, Me-26), 1.68 (s, Me-30), together with the two geminal vinylic protons of the exocyclic methylene group at δ 4.59 (s, H-29b) and 4.70 (s, H-29a), one oxymethine proton at δ 3.60 (dd, *J.*= 11.0, 4.2 Hz), which was assigned to be H-16 and the proton of the C-3 esterified oxymethine group at δ 4.47 (dd, *J.*= 11.9, 5.1 Hz, H-3). The large coupling constants of H-3 (11.0 Hz) and H-16 (11.9 Hz) were indicative of their axial orientations. These data supported the 3β,16β-dihydroxylupeol (calenduladiol) substructure [17]. The presence of a fatty acid ester portion was suggested by the triplet at δ 0.87 (*J.*= 6.8 Hz, H-16`) for one terminal methyl of MeCH_2_-, the broad intensive signal around δ 1.25 ppm for the (CH_2_)*n*, and the doublet triplet at δ 2.28 (*J.*= 7.6, 2.5 Hz, H-2`) for the methylene protons attached to a carbonyl function. The ^13^C-NMR spectrum of **4** exhibiting features characteristic of the lup-20(29)-ene skeleton; two oxygenated methine carbons at δ 77.09 (C-16) and δ 80.55 (C-3), one sp^2^ methylene carbon at δ 109.85 (C-29) and one olefinic quaternary carbon at δ 149.98 (C-20). These ^13^C-NMR data confirmed the calenduladiol substructure of **4** [21,22]. The presence of a fatty acid was supported by the appearance of ^13^C-signals due to an ester carbonyl group at δ 173.76 (C-1`), a long chain of methylene groups at δ 29.18-29.71, and a terminal methyl at δ 14.14 (C-16`). The downfield shift of H-3 (δ 4.47) and C-3 (δ 80.55) signals in **4** compared to the corresponding signals in calenduladiol (δ_H_ 3.17, δ_C_ 79.3) revealed the attachment of the fatty acid at C-3. On the basis of the above spectral data, the structure of compound **4** was identified as calenduladiol-3-*O*-palmitate on the basis of the comparison of its physical and spectroscopic data to those described in the literature [16,17].

Compound (**5**) was isolated as a colorless crystalline mass; mp 160–162 °C. The ESI mass spectrum showed a molecular ion peak at *m*/*z* 413 [M + H]^+^ indicating a molecular formula C_29_H_48_O. Its IR spectrum showed a characteristic absorption band for a hydroxyl group at 3433 cm^−1^ and olefinic bonds at 1645 cm^−1^. The ^1^H-NMR spectrum of compound **5** exhibited the signals of six methyls between δ 0.69 and 1.02, an oxymethine at δ 3.60 (dd, *J.*= 5.1, 11.0 Hz, H-3α) and olefinic protons at δ 5.02 (dd, *J.*= 9.3, 15.3 Hz, H-23), 5.15 (dd, *J.*= 9.3, 15.3 Hz, H-22) and 5.35 (m, H-6) that are characteristic for Δ^5,22^-3β-hydroxy sterols. The ^13^C-NMR spectrum of compound **5** displayed 29 carbon signals of which 6 signals (δ 12.06, 12.27, 18.99, 19.41, 21.08, 21.10) were assigned to 6 methyls, one signal (δ 71.83) for an oxymethine carbon and four signals (δ 121.73, 129.28, 138.34, 140.76) for the four olefinic carbons. Comparison of all these data with those described in the literature confirmed the identity of compound **5** to be a stigmasterol [18,19].

Compound (**6**) was obtained as a colorless oil and had an [M − H]^-^ ion peak at *m/z* 461 in the negative ESIMS, which is compatible with the molecular formula C_29_H_50_O_4_. The IR spectrum of **6** showed absorptions for hydroxyl (3435 cm^−1^), five-membered ring conjugated carbonyl (1720 cm^−1^) and olefinic (1655 cm^−1^) groups. Its UV spectrum showed an absorption band at 236 nm. The ^1^H-NMR spectrum exhibited signals for a hydroxyl proton at δ 4.69 (brs, 4-OH), a methylketone at δ 2.02 (s, H_3_-3a), a tertiary methyl at δ 1.31 (s, H_3_-9a), two olefinic methyls at δ 1.81 (d, *J.*= 0.8 Hz, H_3_-5a) and 1.84 (d, *J.*= 0.8 Hz, H_3_-6a) and four secondary methyls at δ 0.83 (d, *J.*= 5.9 Hz, H_3_-17a), 0.84 (d, *J.*= 6.8 Hz, H_3_-13a), 0.86 (6H, d, *J.*=6.8 Hz, H_3_-21a and H_3_-22). The ^13^C-NMR spectrum of **6** showed 29 carbon signals, including one methyl ketone carbon [δ 24.87 (C-3a)], two vinyl methyl carbons [δ 11.78 (C-5a) and 8.75 (C-6a)], two olefinic carbons [δ 139.51 (C-6) and 163.21 (C-5)], three oxygenated carbons [δ 87.24 (C-9), 89.43 (C-4), and 92.59 (C-2)] and two carbonyl carbons [δ 205.18 (C-1) and 207.01 (C-3)]. The above data suggested that **6** was a spiro compound of two five-membered rings with a branched hydrocarbon chain and was considered to be a α-tocopherol-related compound. The relative stereochemistry of the spiro moiety in **6** was also assigned on the basis of comparison of the chemical shift of H_3_-9a (δ 1.31) of **6** with those of H_3_-9a (δ 1.02) of α-tocospiro A and H_3_-9a (δ 1.29) of α-tocospiro B previously isolated from *Ficus microcarpa* [20]. Therefore, compound **6** was identified as α-tocospiro B.

### 2.2. Anti-Inflammatory Activity

*Pulicaria jaubertii* was used traditionally as a remedy for inflammation [5,7], and several plants of genus *Pulicaria* demonstrated potential anti-inflammatory effect [23,24,25]. In addition, the nature of the *n*-hexane constituents, i.e., triterpenes, which are biologically potential compounds known for their anti-inflammatory, anticancer, and antidiabetic activities [26,27,28] were incentive point to conduct these experiments. However, the current study investigates the anti-inflammatory effect of the *n*-hexane extract of the *Pulicaria jaubertii* for the first time. The anti-inflammatory activity of the total alcoholic and *n*-hexane extracts of *Pulicaria jaubertii* aerial parts was investigated using HRBC membrane stabilization and histamine release inhibition methods and the results were comparable to diclofenac and indomethacin, respectively. The results obtained (Table 1) revealed that both extracts possesses a dose dependent anti-inflammatory activity. The *n*-hexane extract had higher anti-inflammatory potential than the alcoholic extract, which might be attributed to the presence of triterpenes in the *n*-hexane extract. The anti-inflammatory potential by membrane stabilization hypotonic solution-induced hemolysis was found to be higher than that of histamine release inhibition. The *n*-hexane extract was significantly suppressed histamine release with IC_50_ value of 72.9 µg/mL, and showed membrane stabilization activity with IC_50_ value of 60.8 µg/mL. Both membrane stabilization activity and histamine release inhibition contribute to the in-vitro anti-inflammatory activity of the *n*-hexane extract used in our study. 

### 2.3. Anti-Diabetic Activity

The reported antidiabetic activity of the triterpenes [29] encouraged us to investigate the antidiabetic potential of the *n*-hexane extract of *Pulicaria jaubertii*. The anti-diabetic activities of the total alcoholic and *n*-hexane extracts of *Pulicaria jaubertii* aerial parts were in vitro evaluated through determination of their inhibitory effect on alpha amylase and alpha glucosidase enzymes. The result showed that α-amylase and α-glucosidase enzymes were inhibited by both extracts in a dose-dependent manner. However, the total alcoholic extract was more active than the *n*-hexane extract with IC_50_ values of 76.8 to 410.3 µg/mL against α-glucosidase and 192.7 to 498.3 µg/mL against α-amylase, respectively (Table 1). Moreover, both extracts demonstrate lower antidiabetic activity compared to the standard antidiabetic agent, acarbose, which showed IC_50_ value of 34.71 and 30.57 μg/mL against α-amylase and α-glucosidase enzymes, respectively (Table 1).

### 2.4. Cytotoxic Activity

The cytotoxicity of the *n*-hexane extract of *Pulicaria jaubertii* aerial parts was evaluated against three cancer cell lines (HepG-2, MCF7, and PC-3) using cell viability assay. The result (Table 2) showed variable IC_50_ values against the three tested cancer cells ranging from 51.8 to 90.8 µg/mL. The hepatocellular carcinoma (HepG-2) cell line was more sensitive (IC_50_ 51.8 ±3.71 µg/mL) to the *n*-hexane extract followed by the prostate carcinoma (PC-3, IC_50_ 62.2 ± 5.08 µg/mL) cell line and then the breast carcinoma (MCF-7, 90.8 ±5.12 µg/mL), which was the most less sensitive cell line to the extract (Table 2). Moreover, the *n*-hexane extract showed lesser anti-proliferative activity compared to the reported data [10] of the total alcoholic extract of the same plant sample, which showed lower cytotoxic IC_50_ values (24.1 µg/mL, 20.0 µg/mL, and 19.1 µg/mL) against the HepG-2, MCF-7, PC-3 cell lines, respectively.

## 3. Materials and Methods

### 3.1. Materials and Instruments

UV spectra measurements were obtained with a Shimadzu UV-1650PC spectrophotometer; IR spectra were determined with a Shimadzu Infrared-400 spectrophotometer (Shimadzu, Kyoto, Japan). The ^1^H and ^13^C-NMR measurements were carried out on Bruker Avance DRX-850 MHz (for ^1^H) and 212.5 MHz (for ^13^C) (Bruker BioSpin, Billerica, MA, USA) in *CDCl_3_* solution, and chemical shifts were expressed in δ (ppm) with reference to TMS, and coupling constant (*J.*) in Hertz. ESIMS was carried out on Advion compact mass spectrometer (CMS, Ithaca, NY, USA). EIMS was carried on Scan EIMS-TIC, VG-ZAB-HF, X-mass (158.64, 800.00) mass spectrometer (VG Analytical, Inc. Manchester, UK). Open column chromatography was performed on normal-phase Si gel (Si gel 60, Merck, Darmstadt, Germany). Normal-phase and reversed-phase (SPE-C_18_) cartridges (Strata columns) was used for solid phase extraction. TLC was carried out on precoated silica gel 60 F254 (Merck, Darmstadt, Germany) plates. Developed chromatograms were visualized by spraying with 1% vanillin-H_2_SO_4_, followed by heating at 100 for 5 min.

### 3.2. Plant Material

The aerial parts of the plant were gathered from Yemen (Aljar region) in the summer of 2012. It was identified as *Pulicaria jaubertii* by the taxonomist Prof. Dr. Abd El-Rahman Saeed Aldabee from Sana‘a University in Yemen. A specimen’s form of the plant was stored in the herbarium of Faculty of Pharmacy, Al-Azhar University in Cairo under the number of P-01. The plant was dried in shade under room temperature prior to the extraction procedure.

### 3.3. Extraction and Isolation

The powdered air-dried aerial parts of *P. jaubertii* (1.5 kg) were exhaustively extracted with MeOH (7 L × 3) to yield 85 g of a solid extract, which was then suspended in water (500 mL) and successively partitioned with *n*-hexane, ethyl acetate, and *n*-butanol to give 30 g, 12 g, and 8 g, respectively. The *n*-hexane fraction was subjected to VLC on silica gel, eluted with *n*-hexane containing 10% increments of ethyl acetate to give five fractions of A (1.32 g), B (3.13 g), C (1.88 gm), D (5.6), and E (6.2 g). Subfraction A was subjected to si gel CC, eluted with 100% *n*-hexane to give four subfractions of A-1 (100 mg), A-2 (63 mg), A-3 (460 mg), and A-4 (455 mg). Sephadex LH-20 column eluted with MeOH was used to give compound **2** (10mg) from subfraction A-3. Subfraction B was subjected to si gel CC (*n*-hexane:ethyl acetate 100:0-95:5 was used as eluent) to give four subfractions of B-1 (47 mg), B-2 (130 mg), B-3 (90 mg), and B-4 (450 mg). Sephadex LH-20 column eluted with MeOH followed by solid-phase extraction (RP-C_18_) eluted with 95% MeOH in water to 100% MeOH were used to give compound **1** (20 mg) from subfraction B-1. Similarly, compound **4** (10 mg) was obtained from subfraction B-3 by using a column of sephadex LH-20 eluted with MeOH. Subfraction B-4 was subjected to sephadex LH-20 eluted with MeOH followed by Diol CC eluted with *n*-hexane: ethyacetate 95:5 to give compound **5** (5 mg). Subfraction C was subjected to si gel CC, eluted with chloroform to give two subfractions of C-1 (18 mg) and C-2 (754 mg). The subfraction C-1 was subjected to sephadex LH-20 eluted with MeOH to give compounds **3** (5 mg) and **6** (2 mg).

Pseudo-taraxaterol (**1**)**:** A white crystalline solid; IR υ_max_ (KBr) cm^−1^: 3400 (OH), 1650 (C=C); ^1^H-NMR (*CDCl_3_*, 850 MHz) δ 0.69 (brd, *J.*= 9.3 Hz, H-5), 0.73 (s, Me-28), 0.76 (s, Me-24), 0.85 (s, Me-25), 0.95 (s, Me-27), 0.97 (s, Me-23), 0.99 (d, *J.*= 5.9 Hz, Me-29), 1.04 (s, Me-26), 1.63 (brs, Me-30), 3.20 (dd, *J.*= 11.7, 4.5 Hz, H-3), 5.26 (d, *J.*= 6.8 Hz, H-21); ^13^C-NMR (*CDCl_3_*, 212.5 MHz) δ 14.72 (C-27), 15.38 (C-24), 16.03 (C-26), 16.28 (C-25), 17.69 (C-28), 18.28 (C-6), 21.60 (C-11), 21.62 (C-30), 22.53 (C-29), 27.02 (C-15), 27.37 (C-2), 27.62 (C-12), 27.97 (C-23), 34.21 (C-7), 34.38 (C-17), 36.30 (C-19), 36.69 (C-16), 37.07 (C-10), 38.74 (C-1), 38.85 (C-4), 39.20 (C-13), 41.05 (C-8), 42.16 (C-22), 42.32 (C-14), 48.68 (C-18), 50.40 (C-9), 55.27 (C-5), 79.03 (C-3), 118.87 (C-21), 139.86 (C-20); ESI-MS *m\z* 445 [M + H_2_O + H]^+^, *m\z* 425 [M − H]^-^.

Pseudo-taraxasterol acetate (**2**)**:** Colorless needles; IR υ_max_ (KBr) cm^-1^: 1735 (CO), 1642 (C=C); ^1^H-NMR (*CDCl_3_*, 850 MHz) δ 0.73 (s, Me-28), 0.77 (brd, *J.*= 8.0 Hz, H-5), 0.84 (s, Me-24), 0.85 (s, Me-23), 0.87 (s, Me-25), 0.94 (s, Me-27), 0.99 (d, *J.*= 6.8 Hz, Me-29), 1.04 (s, Me-26), 1.63 (brs, Me-30), 2.04 (s, COCH_3_), 4.48 (dd, *J.*= 11.0, 5.1 Hz, H-3), 5.26 (d, *J.*= 7.6 Hz, H-21); ^13^C-NMR (*CDCl_3_*, 212.5 MHz) δ 14.71 (C-27), 16.05 (C-26), 16.38 (C-25), 16.53 (C-24), 17.72 (C-28), 18.20 (C-6), 21.35 (COCH_3_), 21.64 (C-11, C-30), 22.55 (C-29), 23.70 (C-2), 27.03 (C-15), 27.61 (C-12), 27.96 (C-23), 34.17 (C-7), 34.40 (C-17), 36.33 (C-19), 36.70 (C-16), 37.07 (C-10), 38.74 (C-1), 38.85 (C-4), 39.20 (C-13), 41.09 (C-8), 42.18 (C-22), 42.34 (C-14), 48.69 (C-18), 50.34 (C-9), 55.39 (C-5), 81.01 (C-3), 118.89 (C-21), 139.88 (C-20), 171.06 (COCH_3_); EI-MS *m\z* 468 [M]^+^.

β-acetoxytaraxaster-20-en-30-aldehyde (**3**)**:** Colorless needles; UV λ_max_ (MeOH) nm: 235; IR υ_max_ (KBr) cm^−1^: 1733 (CO), 1692, 1645 (C=C-C=O); ^1^H-NMR (*CDCl_3_*, 850 MHz) δ 0.67 (s, Me-28), 0.84 (s, Me-24), 0.85 (s, Me-23), 0.88 (s, Me-25), 0.96 (s, Me-27), 1.02 (d, *J.*= 6.8 Hz, Me-29), 1.03 (s, Me-26), 9.36 (s, H-30), 2.04 (s, COCH_3_), 4.48 (dd, *J.*= 11.9, 5.1 Hz, H-3), 6.71 (dd, *J.*= 6.8, 2.5 Hz, H-21); ^13^C-NMR (*CDCl_3_*, 212.5 MHz) δ 14.69 (C-27), 15.98 (C-26), 16.35 (C-25), 16.52 (C-24), 17.53 (C-28), 18.17 (C-6), 21.35 (COCH_3_), 21.45 (C-11), 23.18 (C-29), 23.69 (C-2), 26.88 (C-15), 27.25 (C-12), 27.99 (C-23), 34.13 (C-7), 34.80 (C-17), 29.38 (C-19), 36.47 (C-16), 37.03 (C-10), 38.44 (C-1), 37.80 (C-4), 39.01 (C-13), 41.05 (C-8), 43.03 (C-22), 42.27 (C-14), 48.20 (C-18), 50.30 (C-9), 55.37 (C-5), 80.96 (C-3), 148.44 (C-20), 149.28 (C-21), 171.07 (COCH_3_), 194.11 (C-30); EI-MS *m\z* 482 [M]^+^.

Calenduladiol-3-*O*-palmitate (**4**): An amorphous white solid; IR υ_max_ (KBr) cm^−1^: 3440 (OH), 1730 (C=O), 1640, 883 (C=C), 725 (long aliphatic chain); ^1^H-NMR (*CDCl_3_*, 850 MHz); calenduladiol δ 0.79 (s, Me-28), 0.83 (s, Me-23), 0.84 (s, Me-24), 0.85 (s, Me-25), 0.98 (s, Me-27), 1.03 (s, Me-26), 1.68 (s, Me-30), 2.49 (m, H-19), 3.60 (dd, *J.*= 11.0, 4.2 Hz, H-16), 4.47 (dd, *J.*= 11.9, 5.1 Hz, H-3), 4.59 (s, H-29b), 4.70 (s, H-29a), fatty acid δ 0.87 (t, *J.*= 6.8 Hz, H-16`), 1.19 (m, H-14`), 1.21 (m, H-15`), 1.25-1.30 (H-4`-H-13`), 1.61 (m, H-3`), 2.28 (dt, *J.*= 7.6, 2.5 Hz, H-2`); ^13^C-NMR (*CDCl_3_*, 212.5 MHz) δ 11.69 (C-28), 16.00 (C-26), 16.17 (C-27), 16.19 (C-25), 16.56 (C-24), 18.19 (C-6), 19.31 (C-30), 20.89 (C-11), 21.30 (C-2`), 23.73 (C-2), 24.74 (C-12), 27.97 (C-23), 29.90 (C-21), 34.18 (C-7), 36.87 (C-15), 37.06 (C-10), 37.25 (C-13), 37.72 (C-22), 37.85 (C-4), 38.41 (C-1), 40.95 (C-8), 44.09 (C-14), 48.61 (C-17), 47.63 (C-19), 47.70 (C-18), 49.92 (C-9), 55.41 (C-5), 77.09 (C-16), 80.55 (C-3), 109.85 (C-29), 149.98 (C-20), fatty acid δ 14.14 (C-16`), 22.71 (C-15`), 25.18 (C-3`), 29.71-29.18 (C-4`-C-13), 31.94 (C-14`), 34.87 (C-2`), 173.76 (C-1`), EI-MS *m\z* 680 [M]^+^.

Stigmasterol (**5**)**:** A colorless crystalline mass; IR υ_max_ (KBr) cm^−1^: 3433 (OH), 1645 (C=C); ^1^H-NMR (*CDCl_3_*, 850 MHz) δ 0.69 (s, Me-18), 0.87 (d, *J.*= 6.8 Hz, Me-27), 0.89 (d, *J.*= 6.8 Hz, Me-26), 0.92 (t, *J.*= 7.6 Hz, Me-29), 1.01 (s, Me-19), 1.02 (d, *J.*= 6.8 Hz, Me-21), 3.52 (hept, *J.*= 5.9 Hz, H-25), 3.60 (dd, *J.*= 5.1, 11.0 Hz, H-3), 5.02 (dd, *J.*= 9.3, 15.3 Hz, H-23), 5.15 (dd, *J.*= 9.3, 15.3 Hz, H-22), 5.35 (m, H-6); ^13^C-NMR (*CDCl_3_*, 212.5 MHz) δ 12.06 (C-18), 12.27 (C-29), 18.99 (C-19), 19.41 (C-26), 21.08 (C-27), 21.10 (C-21), 21.23 (C-11), 24.38 (C-15), 25.42 (C-28), 29.38 (C-16), 31.67 (C-2), 31.94 (C-7, C-8), 36.52 (C-10), 37.26 (C-1), 39.69 (C-12), 40.51 (C-20), 42.23 (C-4), 42.31 (C-13), 45.84 (C-25), 50.16 (C-9), 51.25 (C-24), 55.96 (C-17), 56.87 (C-14), 71.83 (C-3), 121.73 (C-6), 129.28 (C-23), 138.34 (C-22), 140.76 (C-5); ESI-MS *m\z* 413 [M + H]^+^.

α-Tocospiro B (**6**): Colorless oil; UV λ_max_ (MeOH) nm: 236; IR υ_max_ (KBr) cm^−1^: 3435 (OH), 1720 (CO), 1655 (C=C); ^1^H-NMR (*CDCl_3_*, 850 MHz) δ 0.83 (d, *J.*= 5.9 Hz, H-17a), 0.84 (d, *J.*= 6.8 Hz, H-13a), 0.86 (d, *J.*= 6.8 Hz, H-21a, H-22), 1.31 (s, H-9a), 1.62 (s, H-10), 1.70 (m, H-8_β_), 1.77 (m, H-8_α_), 1.81 (d, *J.*= 0.8 Hz, H-5a), 1.84 (d, *J.*= 0.8 Hz, H-6a), 1.88 (m, H-7_α_), 2.35 (m, H-8_β_), 2.02 (s, H-3a), 4.69 (brs, OH-4); ^13^C-NMR (*CDCl_3_*, 212.5 MHz) δ 8.75 (C-6a), 11.78 (C-5a), 19.62 (C-13a), 19.77 (C-17a), 22.41 (C-11), 22.64 (C-22), 22.73 (C-21a), 24.48 (C-19), 24.81 (C-15), 24.87 (C-3a), 25.42 (C-9a), 27.99 (C-21), 32.76 (C-17), 32.81 (C-13), 33.42 (C-7), 36.82 (C-8), 37.30 (C-18), 37.44 (C-14), 37.47 (C-16), 37.48 (C-12), 39.38 (C-20), 42.09 (C-10), 87.24 (C-9), 89.43 (C-4), 92.59 (C-2), 139.51 (C-6), 163.21 (C-5), 205.18 (C-1), 207.01 (C-3); ESI-MS *m\z* 461 [M − H]^-^, 497 [M − Cl]^-^.

### 3.4. Anti-Inflammatory Assay

#### 3.4.1. Membrane Stabilizing Activity

The method was conducted according to the literature [30]. Erythrocyte suspension was prepared by withdrawing blood samples from rats with heparinized syringes through cardiac puncture. Then, phosphate buffer was used to wash blood samples three times with centrifugation after each washing at 3000 rpm for 10 min. Total alcoholic and *n*-hexane extracts in concentrations of 7.81–1000 µg/mL or standard indomethacin were mixed with 0.5 mL of the stock red blood cells (RBCs) suspension and 5 mL of the hypotonic solution consists of sodium phosphate buffer (10 mM) and NaCl (50 mM) adjusted to pH 7.4. Control samples were prepared by similar manner without extracts or indomethacin. Afterward, previous mixtures were withstanding for 10 min in room condition and subjected to 10 min centrifugation at 3000 rpm. Then absorbance was measured for the supernatant at 540 nm and the hemolysis percentage inhibition was calculated from the following equation:

% Inhibition of hemolysis = {A1-A2/A1} × 100(1)

where A1 = Absorbance obtained from blank sample, A2 = Absorbance obtained from blank sample test sample.

#### 3.4.2. Histamine Release Assay

Histamine release effect of total alcoholic and *n*-hexane extracts was measured on U937 human monocytes (ATCC, Manassas, VA, USA). In accordance with literature [31], extracts or standard diclofenac (7.81–1000 μg/mL) were added to U937 cells seeded in a 96-well plate in a concentration of 50,000 in presence or absence of 20 nM Phorbol Myristate Acetate. After 1 h, the treated and untreated cell cultures supernatants were collected and centrifuged at 10,000 rpm for 5 min at 4 °C. Then the samples were evaluated for released histamine by a commercially available EIA kit (SPI-Bio, Montigny le Bretonneux, France). The inhibition in the histamine release was calculated from the following equation:

% Inhibition = (1 − A1/A2) ×100(2)

where, A1 = Absorbance of test, A2 = absorbance of control.

### 3.5. Antidiabetic Activity

#### 3.5.1. Inhibition of Alpha Amylase Enzyme

According to the reported method [32], mixtures consists of 1 mL of phosphate buffer (0.20 mM, pH 6.9) containing 0.5 mg/mL of α-amylase and 1 mL of the total alcoholic and *n*-hexane extracts or standard acarbose in a concentration range of 7.81–1000 μg/mL were incubated at 25 °C for 10 min. One percent of starch solution (1 mL) in sodium phosphate buffer (0.02 M, pH 6.9) was added to the mixtures and incubated for 10 min at room temperature. 3,5-Dinitrosalicylic acid (2 mL) was added and the mixtures were then incubated in a boiling water bath for 5 min. After cooling, 10 mL of distilled water was added to the mixtures. A control test was conducted in a similar manner without test samples. The absorbance was then recorded at 565 nm and the percentage inhibition of amylase was calculated from equation:

% Inhibition = (1−A1/A2) × 100(3)

where, A1 = Absorbance of test, A2 = absorbance of control [33].

#### 3.5.2. Inhibition of Alpha Glucosidase Enzyme

α-Glucosidase inhibitory activity of the alcoholic and *n*-hexane extracts was determined by the method of Shai et al. with minor modification [34]. In 96-well microtitre plates 20 μL of varying concentrations of test samples or acarbose (7.81–1000 μg/mL) were mixed with of phosphate buffer (5 μL of 100 mM, pH 6.8) and α-glucosidase (10 μL of 1 U/mL) (*Saccharomyces cerevisiae*, Sigma Aldrich, Bangalore, India) and preincubated for 20 min at 37 °C. Then, *p*-nitrophenyl-α-D-glucopyranoside (20 μL of 5.0 mM) was added to the mixtures and incubated further for 20 min at 37 °C. The reaction was stopped by adding Na_2_CO_3_ (50 μL of 1 M). Absorbance was recorded at 405 nm to determine the *p*-nitrophenol amount released from the reaction mixture. The reaction mixture without test samples was used as a control where alpha glucosidase enzyme percentage inhibition was calculated from using following equation:

% Inhibition = (1−A1/A2) × 100(4)

where, A1 = Absorbance of test, A2 = absorbance of control [33].

### 3.6. Cytotoxicity Assay

The antiproliferative activity of *n*-hexane extract was conducted against three human tumor cell lines: hepatocarcinoma (HepG-2), breast carcinoma (MCF7), and prostate carcinoma (PC-3) cell lines using MTT assay according to the reported method [35]. All cells were obtained from the American Type Culture Collection (ATCC, Rockville, MD, USA) and were cultured on RPMI 1640 medium supplemented with 10% inactivated fetal calf serum and 50 μg/mL gentamycin. The cells were maintained at 37 °C in a humidified atmosphere with 5% CO_2_ and were sub cultured two to three times a week. Cell viability percentage was calculated by the equation:

Cell viability % = Ac/At × 100(5)

where Ac = Absorbance obtained from of control cells, At = Absorbance obtained from treated cells.

### 3.7. Statistical Analysis

Biological experiments were conducted in independent triplicate, mean were calculated ± St. Error mean. Student’s *t*-test was applied for detecting the significance (*p* < 0.05) of difference between the values of the treated groups and the control. 

## 4. Conclusions

Six compounds, pseudo-taraxaterol, pseudo-taraxasterol acetate, 3β-acetoxytaraxaster-20-en-30-aldehyde, calenduladiol-3-*O*-palmitate, stigmasterol, and α-tocospiro B were isolated using different chromatographic techniques including VLC, silica gel and Sephadex LH-20 columns from *Pulicaria jaubertii n*-hexane extract. The compounds were characterized by the IR, NMR, and MS spectroscopy and by the comparison with literatures. The rare α-tocospiro B was isolated for the first time from family Asteraceae and genus *Pulicaria*, however, all other compounds were recorded for the first time from the plant. The in vitro biological investigation of the plant revealed the significant higher activity of the *n*-hexane extract as an anti-inflammatory agent compared to the total alcoholic extract which showed better antidiabetic and cytotoxic activities. The anti-inflammatory effect of the *n*-hexane extract could be attributed to the extract constituents including triterpenes, which have been prevalently known for this effect. Further animal-based in vivo anti-inflammatory testing of the *n*-hexane extract is an essential future plan to confirm the activity.

## Figures and Tables

**Table 1:** Anti-inflammatory and antidiabetic activities of *Pulicaria jaubertii* alcoholic and *n*-hexane extracts.

Activities	IC_50_ (µg/mL)				
	Extracts		Standards		
	Alcoholic Extract	*n*-Hexane Extract	Diclofenac	Indomethacin	Acarbose
Histamine release inhibition ^[a]^	817.6 ± 1.1	72.9 ±1.7	17.94 ± 1.6	-	
Membrane stabilization ^[b]^	400.3 ± 0.63	60.8 ± 1.3	-	17.02 ± 0.72	
Amylase inhibition ^[c]^	192.7 ± 0.97	498.3 ± 2.1			34.71 ± 1.2
Glucosidase inhibition ^[c]^	76.8 ± 1.5	410.3 ± 1.9			30.57 ± 1.5

All measurements were carried out in triplicate. The IC_50_ value is defined as the concentration of the sample that inhibits 50% histamine release (a), RBCs hemolysis (b), enzyme activity (c) under the assay conditions.

**Table 2:** Cytotoxicity of the *Pulicaria jaubertii n*-hexane extract.

Cell Lines.	IC_50_ µg/mL		Vinblastine **
	*n*-Hexane Extract	Total Alcoholic Extract *	
Hepatocellular carcinoma (HepG-2)	51.8 ± 3.71	24.1	4.6 ± 0.17
Breast carcinoma (MCF-7)	90.8 ± 5.12	20.0	4.6 ± 0.18
Prostate carcinoma (PC-3)	62.2 ± 5.08	19.1	5.5 ± 0.4

* IC_50_ values obtained from the reported data of the total alcoholic extract [10]. All determinations were carried out in triplicate. IC_50_ is defined as the concentration that resulted in a 50% decrease in cell number under the assay conditions. ** Standard anticancer positive control substance.

## Supplementary Materials

The following are available online, Figure S1: ^1^H NMR of compound 1., Figure S2: ^13^C NMR of compound 1, Figure S3: ^1^H NMR of compound 2, Figure S4: ^13^C NMR of compound 2, Figure S5: ^1^H NMR of compound 3, Figure S6: ^13^C NMR of compound 3, Figure S7: ^1^H NMR of compound 4, Figure S8: ^1^H NMR of compound 5, Figure S9: ^13^C NMR of compound 5, Figure S10: ^1^H NMR of compound 6, Figure S11: ^13^C NMR of compound 6.
