# Supplementary material for: Phytochemical Characterization, In Vitro Anti-Inflammatory, Anti-Diabetic, and Cytotoxic Activities of the Edible Aromatic Plant; Pulicaria jaubertii
Source: Molecules. 2021 Jan 3;26(1):203. doi: 10.3390/molecules26010203 (PMC7796184; doi:10.3390/molecules26010203)
Supplement: Supplementary file 1 [file molecules-26-00203-s001.pdf]

Supplementary material

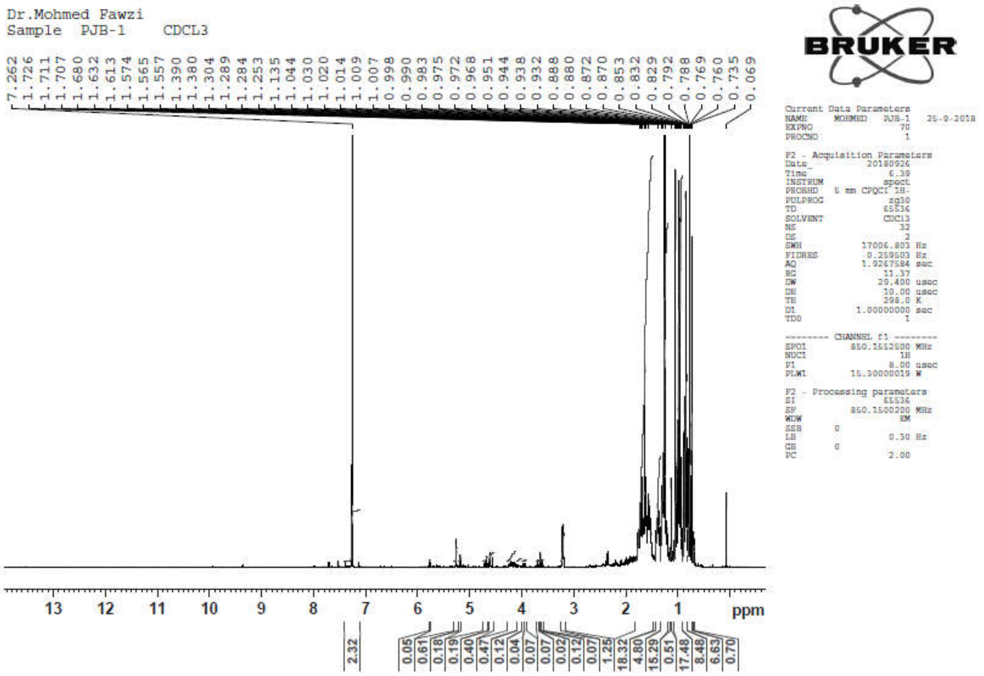

^{1}H NMR of compound 1.

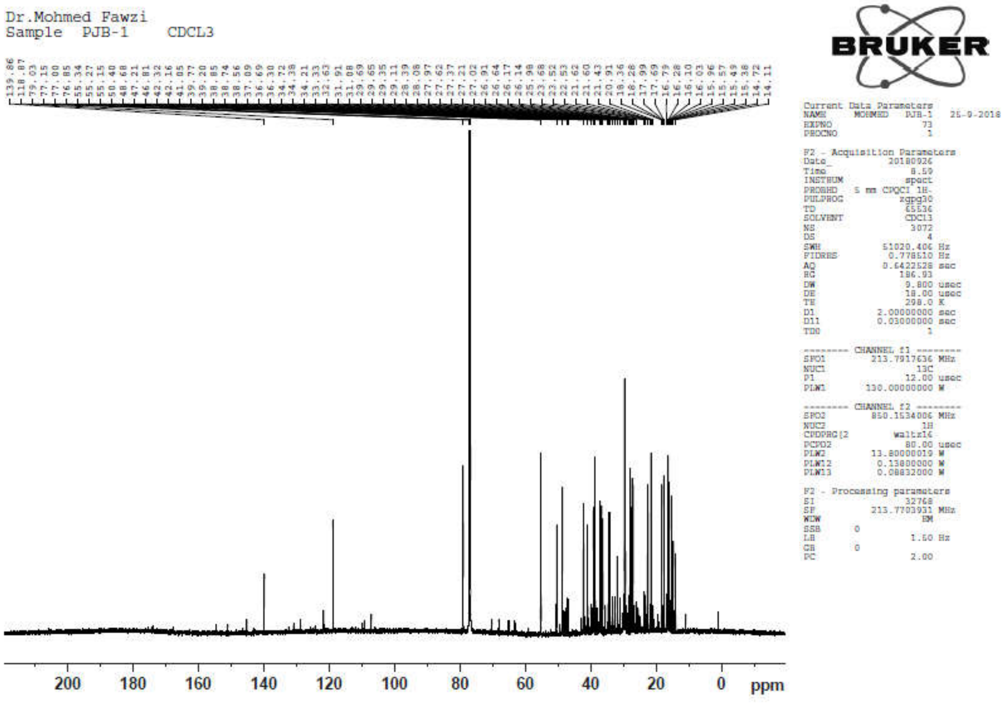

^{13}C NMR of compound 1

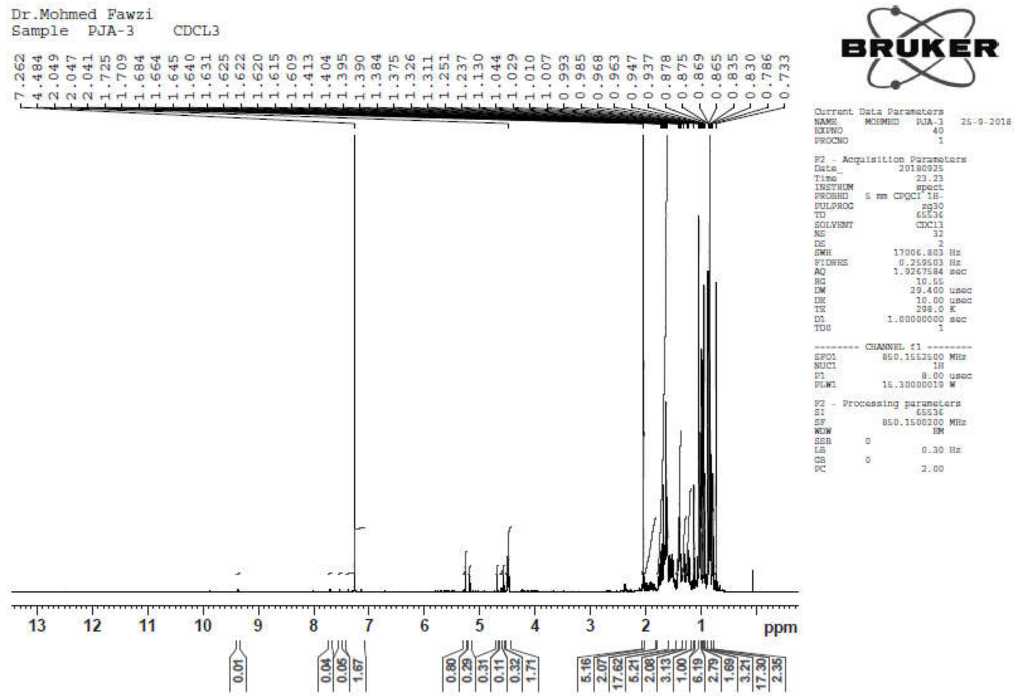

^{1}H NMR of compound 2

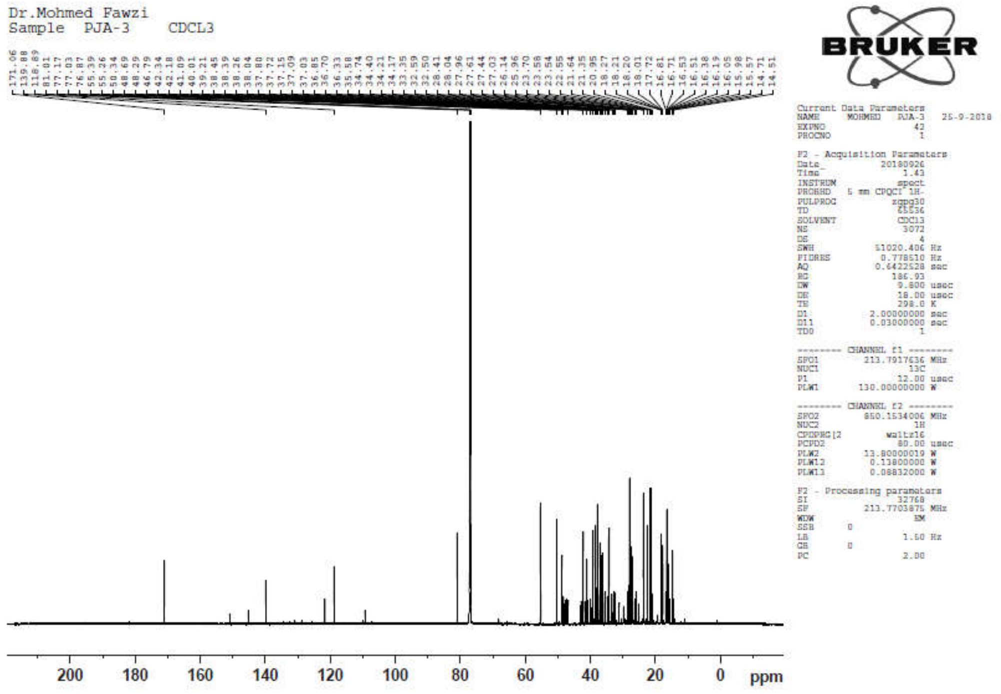

^{13}C NMR of compound 2

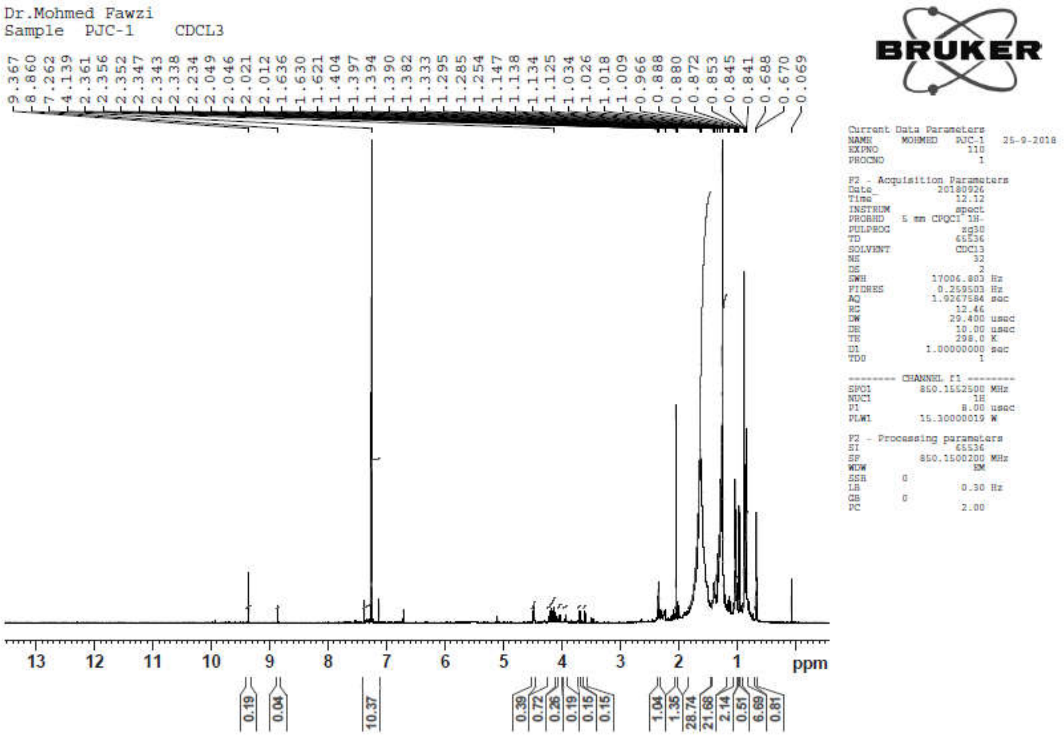

^{1}H NMR of compound 3

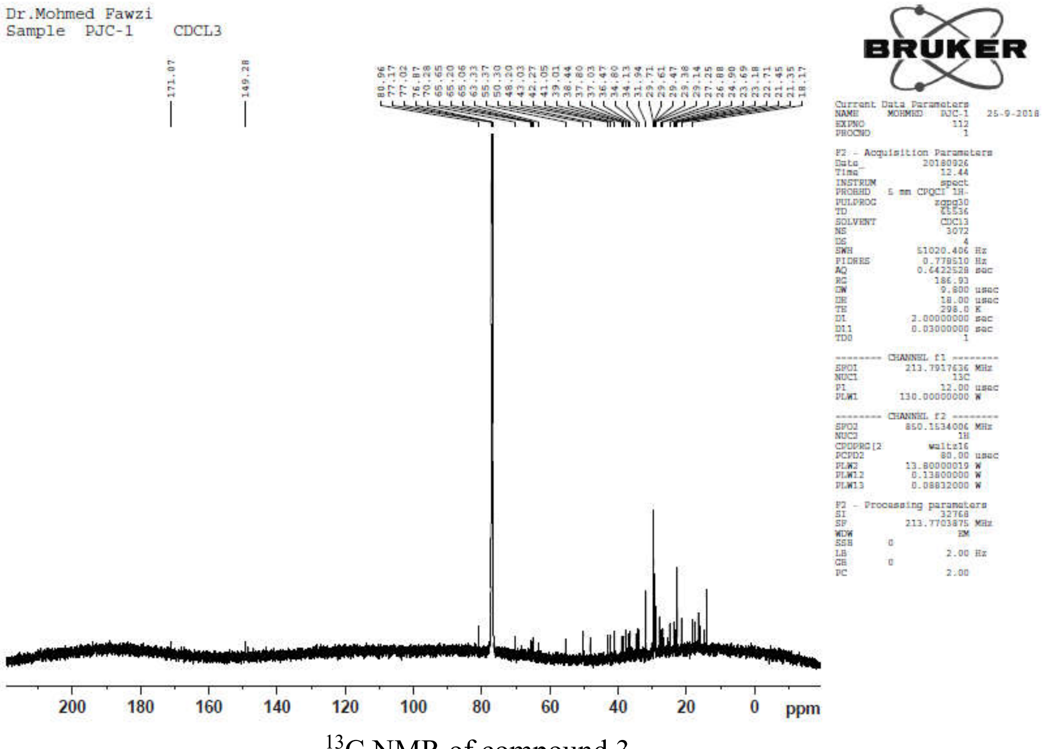

^{13}C NMR of compound 3

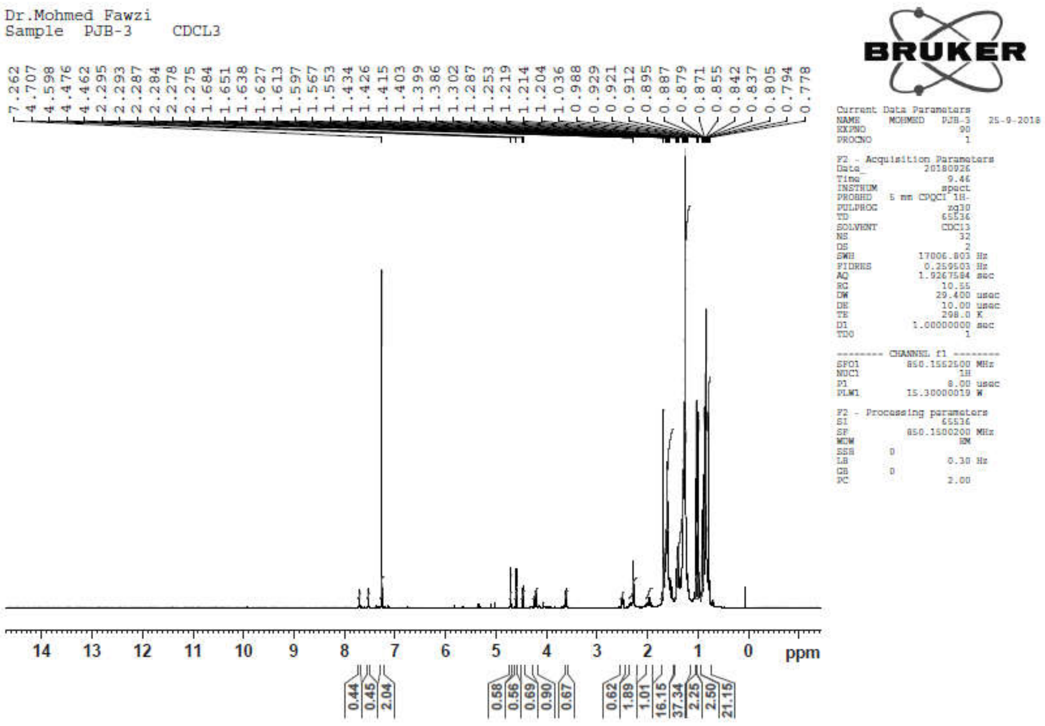

^{1}H NMR of compound 4

^{13}C NMR of compound 4

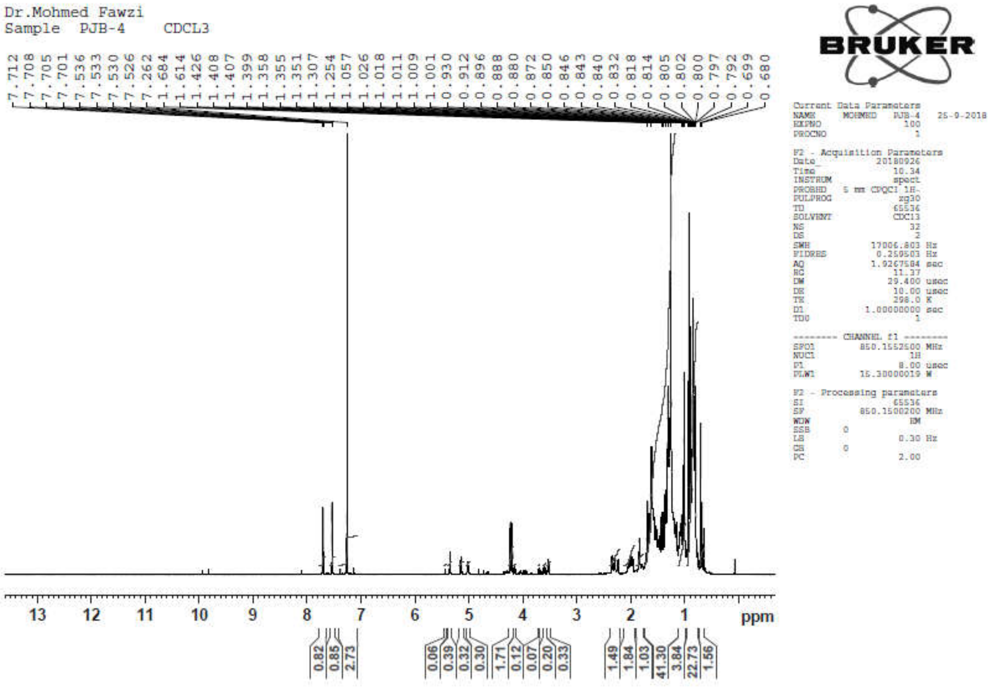

^{1}H NMR of compound 5

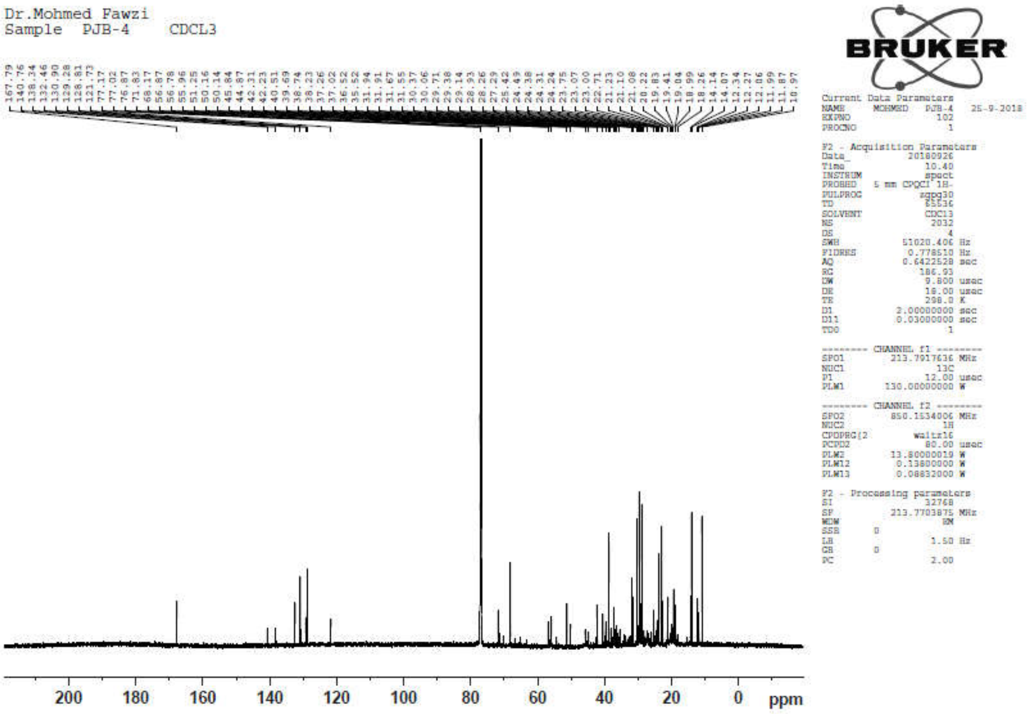

^{13}C NMR of compound 5

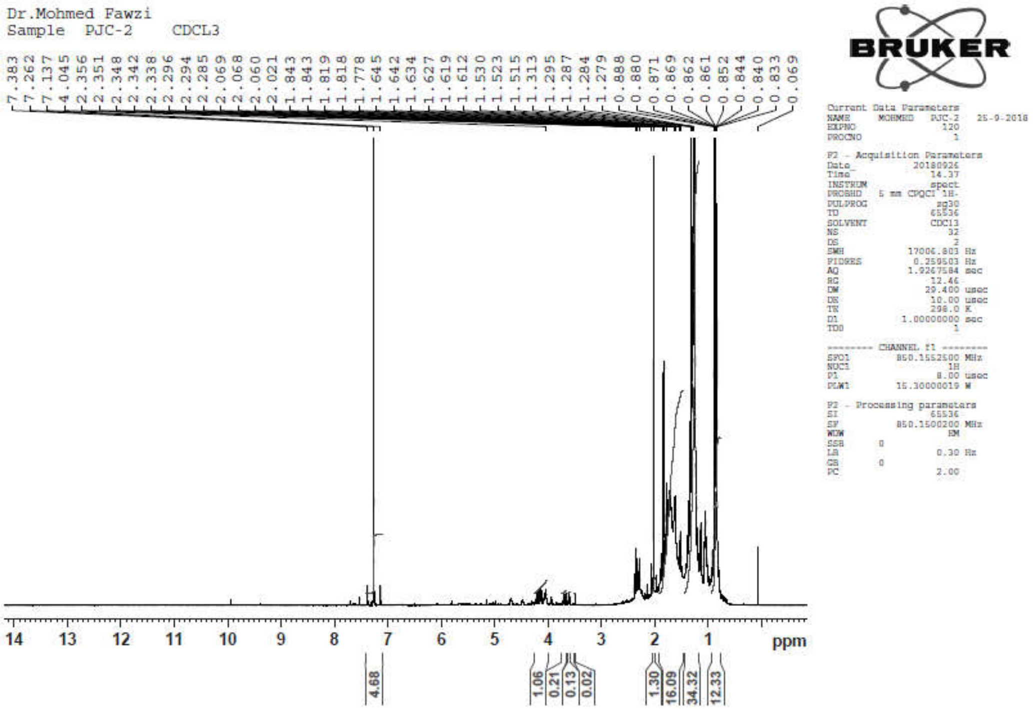

^{1}H NMR of compound 6

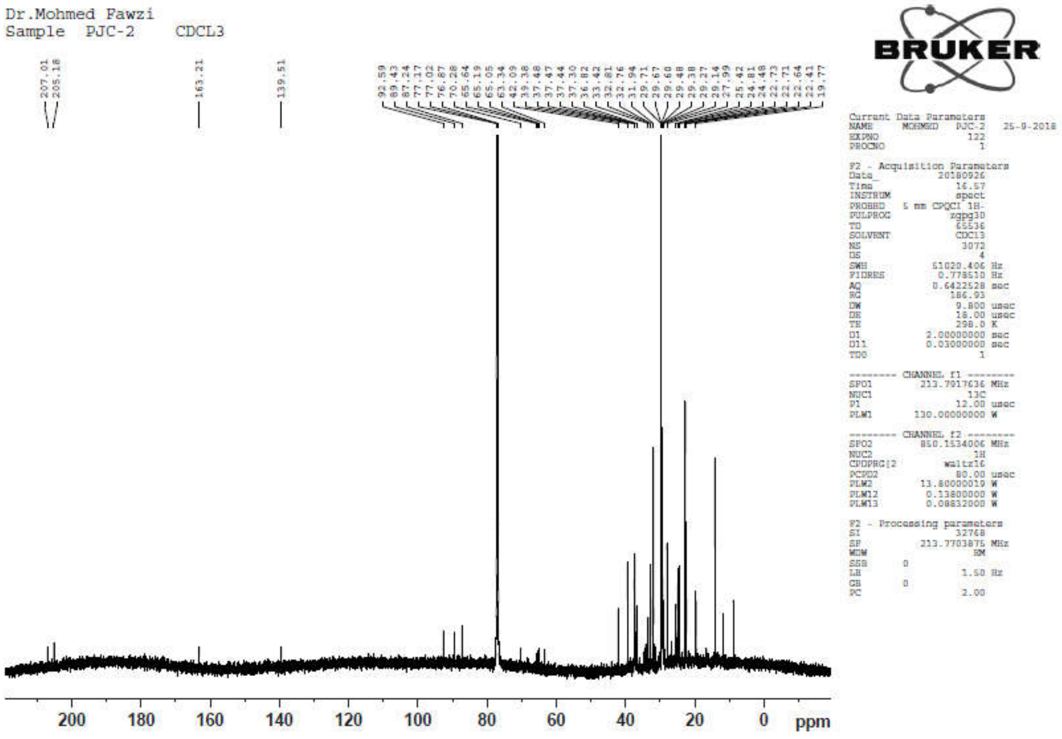

^{13}C NMR of compound 6
